# Development of a collaborative rehabilitation education program for primary care: an educational action research approach

**DOI:** 10.1186/s12875-026-03299-1

**Published:** 2026-03-30

**Authors:** Ryohei Goto, Junji Haruta

**Affiliations:** 1 Institute of Medicine, University of Tsukuba, Tsukuba, Japan; 2 Center for General Medicine Education, Keio University School of Medicine, Tokyo, Japan; 3 Medical Education Center, Keio University School of Medicine, Tokyo, Japan

**Keywords:** Primary care, Physician, Rehabilitation, Educational program, International Classification of Functioning, Disability, and Health, Action research

## Abstract

**Background:**

Primary care clinicians frequently struggle to apply the International Classification of Functioning, Disability, and Health (ICF) in clinical decision-making, despite its relevance for holistic and community-oriented care. To address this gap, we used an educational action research approach informed by participatory principles to iteratively develop an educational program that enables primary care physicians and trainees to understand and apply the ICF in real clinical contexts.

**Methods:**

We employed a multi-cycle educational action research design integrating both quantitative and qualitative data. Each cycle involved an educational session, structured observation, and collaborative reflection. Data sources included questionnaires assessing satisfaction and perceived understanding, open-ended written responses, and follow-up semi-structured interviews with a subset of participants selected based on their open-ended responses. Informants comprised family medicine residents, attending primary care physicians, and medical students. Program materials were refined across cycles by incorporating multimodal resources, including video-recorded patient scenarios and interviews with rehabilitation professionals.

**Results:**

Across three iterative action research cycles (2019–2022), five residents participated in Cycle 1, 38 learners (residents, attending physicians, and medical students) in Cycle 2, and 51 residents in Cycle 3. Lecture-only sessions improved recognition of individual ICF components but did not support understanding of how these elements relate to each other. Introducing video-based patient cases and rehabilitation-therapist interviews helped participants better appreciate the interconnections among environmental factors, activities, and participation. Qualitative feedback indicated that multimodal materials enabled learners to visualize patients’ lived contexts more clearly. In the final iteration, physician-led explanations of clinical observation points made the content feel more applicable to routine clinical practice. Across sessions, 75–90% of respondents reported high satisfaction and greater confidence in using the ICF framework.

**Conclusion:**

Through an iterative educational action research process, we developed a multimodal education program that supported primary care clinicians in understanding the ICF framework and recognizing its relevance to clinical practice, addressing gaps left by lecture-only training. Multimodal and contextually grounded educational materials could potentially foster the application of ICF perspectives in primary care.

**Supplementary Information:**

The online version contains supplementary material available at 10.1186/s12875-026-03299-1.

## Background

As the global population continues to age and healthcare expenditure rises alongside economic development [1], sustaining effective healthcare delivery has become a major challenge worldwide. Many health systems have emphasized the importance of shifting from hospital-centered care toward community-based, person-centered care models [2, 3]. Japan faces this challenge acutely, as the country is undergoing one of the fastest demographic transitions toward a super-aged society [4]. While Japan currently possesses healthcare resources (e.g., hospital beds and healthcare professionals) comparable to those of other high-income nations [5], future projections indicate a growing discrepancy between the availability of resources and the population’s healthcare needs [6]. Thus, the Japanese government has launched the “Community Healthcare Vision” [7], which aims to reallocate hospital functions in line with anticipated needs. Specifically, it forecasts a surplus of acute care beds and shortage of recovery-phase beds that support rehabilitation and home transition [8]. As part of this structural reform, a community-based integrated care system has been promoted to deliver comprehensive services, including medical, nursing, and preventive care; housing; and social support at a local level [9, 10]. Within this new system, primary care is expected to play a pivotal role in coordinating holistic, community-based services [11].

Primary care physicians frequently care for patients with complex conditions such as multi-morbidities and polypharmacy [12–15], which are closely linked to functional decline and increased risk of disability [16]. The global trend has reinforced the need for clinical approaches grounded in the biopsychosocial model [17] and for integrating rehabilitation perspectives within primary care. Rusk described rehabilitation as a core component of primary care [18], underscoring the importance of understanding patients’ lived environments and establishing meaningful and personalized care goals. However, even though ICF has been promoted internationally as a unifying biopsychosocial framework, a recent global survey showed that its use remains non-mandatory in many countries, and substantial variation exists in how it is implemented in clinical practice and policy settings [19]. These findings highlight persistent challenges in inter-contextual application, partly due to difficulties in understanding and operationalizing the dynamic interactions among ICF components.

In Japan, family physicians certified by the Japan Primary Care Association (JPCA) are trained under a postgraduate program accredited by the World Organization of Family Doctors [20]. This program includes a comprehensive four-year curriculum with multiple assessments, including written, clinical, portfolio, and oral components [21]. The portfolio requires case-based reflections across 20 domains (e.g., chronic disease care, multi-morbidity, and patient-centered care) [22]. One of the required portfolio domains is “disability and rehabilitation,” which evaluates trainees’ ability to assess patients using the International Classification of Functioning, Disability, and Health (ICF) framework [23], formulate rehabilitation goals and prescriptions, and reflect on the appropriateness of their interventions. Despite these requirements, many trainees and practicing physicians, especially those trained predominantly under biomedical paradigms, struggle to grasp and apply the concepts of disability and rehabilitation in clinical settings [18]. Although previous studies examined the current state of rehabilitation training in residency programs in Japan [24], there remains a lack of clinically relevant and internationally comparable educational programs designed to support the practical and context-sensitive use of the ICF framework in primary care.

This study aimed to develop a collaborative rehabilitation education program that enables primary care physicians to apply the ICF perspective in outpatient and home visit settings. By promoting a multidimensional and integrative understanding of patients’ lives, this program seeks to enhance the quality of community-based care and the evolving role of primary care physicians in Japan’s integrated care system.

## Methods

### Design

This study employed an educational action research approach informed by participatory principles, which aims to facilitate practice improvement while generating transferable knowledge through iterative cycles of implementation and reflection [25]. We adopted a framework informed by the spiral model proposed by Kemmis and McTaggart, conceptualizing the process as repeated cycles of planning, action, observation, and reflection [26]. In each cycle, we implemented an educational activity, observed learners’ responses, and collected quantitative and qualitative data to inform reflection and refinement of subsequent cycles. Participants contributed primarily through structured feedback (questionnaires and open-ended responses), interviews, and informal discussions that informed program revisions; however, they did not engage in formal co-analysis or co-interpretation of the qualitative data. These cycles were repeated and adapted to evolving contexts [27]. This iterative process was used to develop and refine a rehabilitation education program between September 2019 and December 2022.

### Overview of action research cycles

This educational action research process was conducted in three iterative cycles, each designed to enhance the primary care physician’s ability to apply the ICF framework in clinical settings. An overview of each cycle’s goal, activities, and participants is summarized in Table 1, including clinical experience and training level for each participant group. Age and gender were not collected, as the primary focus was on educational needs and learning processes rather than sociodemographic factors.

**Table 1:** Summary of AR cycles

Cycle	Main Goal	Key Activities	Participants
Cycle 1	Assess residents’ ICF readiness and deliver foundational education	Clinical observation, 20 min ICF lecture per resident	Five residents (postgraduate years 3–6)
Cycle 2	Introduce video-based and inter-professional perspectives	Case videos, group discussion, summary lecture	Eight physicians (8–25 years in practice), seven residents (postgraduate years 3–6), 23 medical students (years 1–6)
Cycle 3	Enhance clinical relevance through physician-led examples	Pre-lecture explanations by physicians, reflective discussion	51 residents (postgraduate years 3–6)

*ICF* International Classification of Functioning, Disability, and Health, *min* minutes

### Setting and participants

The educational program was initially developed within the Tsukuba Family Medicine and Hospital General Medicine Program (Tsukuba Program) operated by General Medicine and Primary Care at the University of Tsukuba Hospital, where the authors are affiliated. This program is a four-year postgraduate family medicine residency that includes rotations through inpatient, outpatient, and home care settings. The initial implementation targeted family medicine residents and attending primary care physicians in the Tsukuba Program.

Subsequently, the educational program was expanded to include medical students, resident physicians, and supervising physicians at educational events hosted by the Japanese Society of Primary Care, including the Family Medicine Summer Seminar, Autumn Continuing Education Seminar, and a workshop organized by the Resident Physicians Committee. Each iteration of the program was revised based on participant feedback and reflection.

Participants were family medicine residents, attending primary care physicians, and medical students who voluntarily took part in the educational sessions associated with each AR cycle. Because the program was embedded within ongoing educational activities, no exclusion criteria were applied and sample size was not predetermined or calculated statistically. This study used a purposive and convenience sampling strategy. A total of five residents participated in Cycle 1; 38 participants (seven residents, eight attending physicians, and 23 medical students) in Cycle 2; and 51 residents in Cycle 3.

## Sampling and recruitment

Because this study was conducted as part of ongoing educational activities using an educational action research approach, the sample size was not predetermined or statistically calculated. Participants comprised family medicine residents, attending primary care physicians, and medical students who voluntarily participated in the educational sessions associated with each action research cycle. No exclusion criteria were applied.

The selection of participant groups and settings was guided by a conceptual rationale aligned with the aims of educational action research, which emphasizes iterative development, stakeholder engagement, and responsiveness to learners’ needs. The ICF framework is inherently multidimensional and intended to support clinical reasoning across different stages of medical training and professional practice in primary care. Therefore, including medical students, residents, and attending primary care physicians enabled us to capture diverse perspectives from learners and practitioners at varying levels of experience, allowing identification of differences in learning needs, conceptual challenges, and clinical applicability of the ICF. The Tsukuba family medicine training program and national educational seminars were selected as appropriate settings because they represent complementary real-world educational contexts in which primary care clinicians are expected to integrate rehabilitation perspectives into routine practice. The training program provided opportunities to explore the introduction of ICF concepts in structured postgraduate education, while the national seminars reflected continuing professional development contexts involving more experienced clinicians. This combination of participant groups and educational settings was essential for iteratively developing and refining an ICF education program that addresses learners’ conceptual understanding while remaining applicable to everyday clinical practice in primary care.

### Research team

The research team comprised two professionals with complementary expertise. RG is a male physical therapist with a PhD and extensive experience in quantitative and qualitative research, who served as a faculty member responsible for resident education within the Tsukuba Program. JH is a general practitioner trained in qualitative methods and experienced in rehabilitation collaboration, who served as faculty and supervisor in the same program until 2020. RG and JH have worked together at the University of Tsukuba for four years and have collaborated on research and educational activities. This research initiative was born out of their shared interest in improving rehabilitation education for primary care, and they jointly conducted all aspects of program development and evaluation.

### Data collection and analysis

After each implementation cycle, participants were invited to complete a web-based questionnaire, administered via an online Google Form. The survey included both multiple-choice items (e.g., program satisfaction, understanding of content) and open-ended questions to assess participants’ understanding of the ICF framework and the perceived effectiveness of the educational program. The questionnaire was developed specifically for this study through iterative discussions between the two authors, based on the learning objectives and intended outcomes of the educational program. Because the questionnaire was developed to support iterative refinement across cycles within this educational action research approach, it was not pilot-tested or formally validated. The English version of the questionnaire is provided as a Supplementary File (Supplementary 1).

Follow-up semi-structured interviews were conducted with a subset of participants whose open-ended responses demonstrated rich, reflective, or contextually detailed insights that could inform program refinement. Although responses indicating difficulty with ICF application were also reviewed, most interviewees were those who provided elaborate reflections rather than those expressing difficulty alone. Feasibility and rapport were also considered, and interviews were requested only from participants with whom the researchers had an established educational or working relationship. To reduce potential bias, we avoided selecting only strongly positive or negative responses and ensured representation across different training stages. A semi-structured interview guide was used for these interviews, which was developed through iterative discussions between the two authors, based on the learning objectives of the educational program and the competencies expected of primary care physicians when applying rehabilitation perspectives in clinical practice. The full interview guide is provided as Supplementary File (Supplementary 2).

To evaluate the outcomes of each action research cycle, a logic model was constructed to map inputs, activities, outcomes, and intended impact [28]. Analyses were conducted through iterative discussions between the authors, drawing on data from surveys, interviews, reflections, and field notes which were maintained throughout the iterative educational action research process. The logic model framework was guided by the ICF structure, which organizes human functioning and disability into six components: health status, body functions and structures, activities, participation, environmental factors, and personal factors [29]. This framework enabled a multifaceted analysis of the educational program’s effectiveness.

In this educational action research approach, quantitative and qualitative data were not combined through formal mixed-methods integration. Instead, they were used complementarily within each action–observation–reflection cycle. Quantitative data provided a broad assessment of participants’ satisfaction and perceived understanding, while qualitative data (open-ended responses, interviews, and field notes) offered explanatory insights that guided revisions to subsequent cycles. Although participants did not engage in formal co-analysis of qualitative data, their perspectives played a central role in the iterative program refinement process. Feedback obtained through open-ended responses, interviews, and informal discussions was incorporated into each cycle during the reflection phase. This form of participant involvement is consistent with educational action research, where learners contribute to iterative refinement of the program rather than performing collaborative coding or thematic analysis.

Several strategies were employed to ensure trustworthiness of the study within a qualitative educational action research approach informed by participatory principles. Credibility was strengthened through triangulation of multiple data sources, including questionnaires, open-ended written responses, semi-structured interviews, and reflective field notes. Dependability was supported by maintaining detailed documentation of decisions, observations, and reflections throughout each action research cycle. Confirmability was enhanced through collaborative analysis by two researchers with different professional backgrounds. Transferability was addressed by providing rich descriptions of the educational context, participant characteristics, and iterative development processes. Together, these strategies helped ensure methodological rigor appropriate to educational action research.

## Results

### Preliminary assessment

As part of their training, Tsukuba Program residents are required to submit portfolios reflecting on their clinical experiences across designated competency domains. Upon reviewing portfolios in the “disability and rehabilitation” domain, the research team established that while residents could describe individual ICF components, they struggled to interpret the interrelationships among these elements and integrate them into real-life patient contexts.

### Cycle 1: Foundational understanding of the ICF

#### Plan

The researchers hypothesized that residents lacked a comprehensive understanding of the interactive nature of ICF. To address this, they delivered lectures using the ICF framework. However, as RG had limited insight into the residents’ clinical contexts, he first observed their home visits before conducting lectures.

#### Action

Between September 2019 and March 2020, RG observed the clinical practice of each resident enrolled in the Tsukuba Program. During home visits, residents typically began with greeting patients and their families and engaging in brief conversations, followed by checking vital signs. Most of the visit was focused on medical management, such as symptom assessment and medication review. Although residents occasionally asked whether patients had any difficulties in daily life, they rarely posed specific questions about daily activities. Following these observations, each resident received a 20-minute lecture on the ICF components and their interrelationships. Training was conducted individually (one resident per day) at different regions and facilities, with five residents participating over five days.

#### Observation

Residents who received ICF lectures reported that their understanding of ICF concepts had improved. However, their subsequent portfolios did not demonstrate an understanding of how the ICF components interact with one another in relation to patients’ life contexts. RG and JH concluded that although the lectures helped clarify individual ICF components, they were insufficient to improve the overall portfolio quality.

#### Reflection

After reviewing the process, RG and JH determined that simply delivering lectures on ICF concepts was insufficient. To foster practical understanding, it was necessary to demonstrate how these concepts could be applied to real-life clinical cases that residents encounter. They also recognized the importance of incorporating perspectives of rehabilitation therapists, which are often underrepresented in medical training. RG further reflected on the need for residents to develop a deeper appreciation of the rehabilitation perspective. The logic model derived from Cycle 1 is presented in Table 2.

**Table 2:** Logic model after Cycle 1

Input	Activity	Output	Outcome	Impact
- Human Resources: Physical therapist, Supervising physicians (primary care physicians), residents - Materials: Laptop, handouts - Funding: JSPS KAKENHI	− 20 min lecture on ICF components and related factors - One-on-one implementation following clinical observation	- Conducted with five residents - Consideration of ICF components and related factors in actual patient cases	- Understanding of the ICF framework	- Improved quality of the “Disability and Rehabilitation” portfolio

*ICF* International Classification of Functioning, Disability, and Health, *min* minutes

### Cycle 2: Online program implementation

#### Plan 2

RG and JH created video teaching material featuring simulated patients in clinical settings and conducted interviews with rehabilitation therapists. These resources were designed to help residents visualize specific cases and better understand the interrelationships between ICF components from a rehabilitation perspective. Two versions of the clinical scenario videos, outpatient care and home visit care, were created to suit varying levels of learners’ readiness.

The videos not only illustrated the ICF components and their interconnections but also demonstrated effective interview techniques grounded in the ICF perspective. Videos featuring rehabilitation therapists included recorded interviews and scenes from actual home rehabilitation visits, showcasing how therapists interpret patients’ lives through their professional perspective. An educational program was designed to incorporate these videos into individual and group learning activities. Considering the COVID-19 pandemic, the program was adapted for online delivery.

#### Action 2

The program began with a lecture on the ICF framework, followed by viewing clinical scenario videos. Participants conducted individual assessments using the ICF, shared their evaluations and engaged in group discussions. After watching rehabilitation therapist videos, they repeated the individual assessments and group discussions. A final summary lecture was delivered by RG, conducting the 90-minute workshop. Following discussions between RG and JH, the program’s primary objective was redefined from improving portfolio quality to enhancing clinical applicability. The target participants were expanded to include not only residents but also attending physicians and medical students. The revised program was implemented with seven residents and eight supervising physicians in June 2022, and later with 23 medical students at the Family Medicine Summer Seminar in August 2022. In the student sessions, both rehabilitation therapists and primary care physicians acted as facilitators in each group.

#### Observation 2

During group work, participants exchanged ideas and received additional input from the facilitators. Many deepened their understanding of how elements such as “environmental factors” influence “activities” and “participation” within the ICF framework.

Post-program surveys revealed that approximately 80% of the participants were satisfied with the program content. Feedback included: “Watching the therapist’s video after the doctor’s video helped me better understand the differences in perspectives between professions” (third-year medical student), and “I was able to gain a more multifaceted perspective” (fourth-year medical student).

#### Reflection 2

RG and JH reflected that combined use of physician and rehabilitation therapists videos helped participants understand the interplay among ICF components. However, they identified a remaining gap: whether primary care physicians could effectively apply the ICF perspective in clinical practice. They considered that concrete examples from physicians might further enhance practical understanding. The updated logic model for Cycle 2 is presented in Table 3.

**Table 3:** Updated logic model after Cycle 2

Input	Activity	Output	Outcome	Impact
- Human Resources: Physical therapist, Supervising physicians (primary care physicians), residents, simulated patients, video production crew - Materials: Laptop, handouts, Zoom, video materials - Funding: JSPS KAKENHI	- Conducting a 90 min workshop titled “How can outpatient/home visits uncover the lives of patients and families?” - Lecture on ICF components and related factors - Viewing clinical scenarios on video - Individual evaluation using ICF framework and group sharing - Viewing videos of home rehabilitation sessions - Group discussion on ICF-based evaluations - Summary lecture by a physical therapist	- Workshop conducted with eight supervising physicians, seven residents, and 23 medical students - Participants evaluated ICF components and related factors in actual cases - Participants gained perspectives of both physicians and rehabilitation professionals - Participants grasped interactions among ICF components based on rehabilitation perspectives	- Understanding of the ICF framework - Understanding the perspectives of home rehabilitation therapists - Understanding the interactions among ICF components and related factors	- Primary care physicians can apply the ICF framework in clinical practice

### Cycle 3: Clinical application program

#### Plan 3

To provide concrete examples of how to apply the ICF framework in practice, the program was further refined. In this version, participants were encouraged to not only understand ICF concepts and their interrelationships but also share their observations from patient’s home environments and behaviors which are critical elements for grasping patients’ life experiences and values. Additionally, it was believed that these examples would be relatable and applicable if explained by physicians rather than other professionals from other disciplines. Therefore, explanatory input from primary care physicians was added.

#### Action 3

Prior to the final summary lecture by physical therapists, physicians provided explanations on key clinical observation points relevant to ICF-based assessment. This updated version of the program was implemented as an online seminar organized by the JPCA, with 51 residents participating between September and December 2022.

#### Observation 3

Participants reported enhanced understanding of ICF application in clinical practice. Selected comments included: “Using videos, I was able to understand the ICF visually; although I focused on creating portfolios, I learned about the practical applications of the ICF in real clinical settings” (fifth-year physician); “Through discussions and presentations by other groups, my understanding of how to apply ICF concepts in clinical practice deepened” (fifth-year physician); and “Continuous learning is necessary to apply ICF concepts effectively in clinical practice” (sixth-year physician).

These reflections indicated growing confidence and the need for ongoing education.

#### Reflection 3

The addition of physician-led clinical observation points supported more realistic and applicable learning. Participants were better able to envision how to use ICF in their own practice. The final logic model from Cycle 3 is presented in Table 4.

**Table 4:** Updated logic model after Cycle 3

Input	Activity	Output	Outcome	Impact
- Human Resources: Physical therapist, Supervising physicians (primary care physicians), residents, simulated patients, video production crew - Materials: Laptop, handouts, Zoom, video materials - Funding: JSPS KAKENHI	- Conducting a 90 min workshop titled “How can outpatient/home visits uncover the lives of patients and families?” - Lecture on ICF components and related factors - Viewing clinical scenarios on video - Individual evaluation using ICF framework and group sharing - Viewing videos of home rehabilitation sessions - Group discussion on ICF-based evaluations - Explanation of clinical observation points by a physician to facilitate real-world application - Summary lecture by a physical therapist	- Workshop conducted with eight supervising physicians, seven residents, and 23 medical students - Participants evaluated ICF components and related factors in actual cases - Participants gained perspectives of both physicians and rehabilitation professionals - Participants grasped interactions among ICF components based on rehabilitation perspectives - Envisioned practical applications of the ICF through clinical scenarios	- Understanding of the ICF framework - Understanding the perspectives of home rehabilitation therapists - Understanding the interactions among ICF components and related factors - Understanding how to apply the ICF in actual clinical practice	- Primary care physicians can apply the ICF framework in clinical practice

## Discussion

In this study, we developed and refined an educational program through an iterative educational action research approach informed by participatory principles to support primary care physicians in applying the ICF framework in clinical practice. In the initial stage, family medicine residents demonstrated an understanding of individual ICF components but struggled to grasp the interrelationships among these elements or integrate them with patients’ life contexts. To address this gap, we created video materials simulating clinical scenarios and conducted interviews with rehabilitation professionals. These additions enabled learners to visualize how different ICF components interact in reality. To further support clinical application, the program was revised to include examples presented by practicing physicians. This addition helped participants better conceive how the ICF framework could be implemented in their own clinical practice. Regarding medical education, where rehabilitation is often underrepresented, this program offers a practical and scalable model for both medical students and residents.

As suggested by participants’ feedback and data from earlier iterations, paper-based ICF materials do not sufficiently convey the dynamic interrelationships between ICF components. In contrast, our video-based program allowed learners to experience ICF as a flexible, context-dependent framework. This aligns with earlier findings demonstrating that video case-based learning can enhance learner’s ability to adopt patient- and family-centered approaches and more effectively consider psychosocial dimensions compared to text-based cases [30]. Additionally, it was demonstrated that presenting cases through video may promote cognitive and metacognitive processes in group work more than presenting cases through text [31]. Participants in our program reported that the visual format helped them interpret the ICF components in an integrated and contextually grounded manner.

A distinguishing feature of this program is its intentional integration of perspectives across and within professions, specifically, physical therapists and primary care physicians, as well as learners at various career stages, including medical students, residents, and practitioners. Participants described the learning environment as rich and multifaceted, largely due to the integration of different professional and learner perspectives. Interprofessional learning has been shown to enhance participants’ self-efficacy and role awareness [32]. Our findings are consistent with Selman’s theory of social cognition development, which highlights the importance of understanding others’ perspectives in learning [33], and Khalili’s interprofessional socialization framework, which describes how exposure to diverse professional perspectives fosters both inter-professional and professional identity formation [34]. In our program, physicians directly engaged with rehabilitation therapists to understand their perspectives and then observed how such insights were applied by peers in similar clinical roles [35–37]. This back-and forth between “other professional perspectives” and “own practice” helped deepen participants’ understanding of their responsibilities in community-based integrated care. Recognizing “assessment of functional living” as part of the primary care physician’s role reflects a meaningful shift in professional identity.

We consider this educational approach potentially transferable, as its focus on contextualized understanding of patient functioning is relevant across multiple professional roles in community-based integrated care. Its emphasis on understanding patients’ life contexts and using multifaced evaluation aligns with the needs of various professionals in community-based integrated care systems. Given the increasing importance of collaboration among health, nursing, and welfare services in aging societies, the program’s ICF based, video-supported, group discussion format can be adapted to nursing, rehabilitation, and inter-professional training across different settings. Its online delivery further enhances its scalability and accessibility across regions and institutions.

### Limitations

This study has several limitations. Although this study was informed by participatory principles, participant involvement did not extend to shared ownership of inquiry or formal co-analysis of data. Accordingly, this study is best understood as educational action research informed by participatory principles rather than a fully participatory action research study. In addition, the evaluation relied primarily on self-reported measures such as satisfaction and perceived understanding. While suitable for the early stages of program development, these indicators do not provide evidence of actual behavioral change or application of the ICF framework in clinical practice. Therefore, improvements should be interpreted as perceived rather than objectively demonstrated. Future studies should incorporate objective or longitudinal assessments to examine whether changes in conceptual understanding translate into clinical behaviours. Moreover, participants varied in training stage and clinical experience, including medical students, residents, and attending physicians. Given the heterogeneity of learner levels, interpretations of the program may vary. Future studies should examine outcomes by training level.

## Conclusions

This study developed and iteratively refined a rehabilitation education program for primary care physicians using an educational action research approach informed by participatory principles. By integrating video-based case scenarios and perspectives from both rehabilitation professionals and primary care physicians, the program promoted a deeper understanding of the ICF framework and its practical relevance in patient care. The combination of profession-specific and inter-professional content supported repeated engagement with conceptual and applied learners’ self-efficacy. These findings suggest that iterative engagement with both conceptual and applied resources is essential for fostering meaningful use of the ICF in primary care practice.

## Supplementary Information

Supplementary Material 1.

Supplementary Material 2.

## Data Availability

Anonymized questionnaire data collected from participants during the action research process are not publicly available, but are available from the corresponding author upon reasonable request, in accordance with ethical guidelines.The English version of the questionnaire and the semi-structured interview guide used in this study is provided as a Supplementary File.

## Abbreviations

ICF: International Classification of Functioning, Disability, and Health
JPCA: Japan Primary Care Association
