# Supplementary material for: Development of a collaborative rehabilitation education program for primary care: an educational action research approach
Source: BMC Prim Care. 2026 Mar 30;27:184. doi: 10.1186/s12875-026-03299-1 (PMC13154494; doi:10.1186/s12875-026-03299-1)
Supplement: Supplementary file 1 — Supplementary Material 1. [file 12875_2026_3299_MOESM1_ESM.docx]

# English Version of the Questionnaire

Title: Survey on the educational program using the ICF framework

Q1. How would you rate your satisfaction with today’s educational session?

- Very satisfied
- Satisfied
- Somewhat dissatisfied
- Dissatisfied

Q2. How well do you think you understood the content of today’s session?

- I understood it very well
- I understood it fairly well
- I didn’t understand it very well
- I didn’t understand it at all

Q3. Please share any comments, impressions, or suggestions you have about the program.

(Free text)

Q4. Please describe any areas for improvement in today’s educational program.

(Free text)

Q5. If you have any other comments or feedback, please feel free to share them below.

(Free text)
