# Supplementary material for: Development of a collaborative rehabilitation education program for primary care: an educational action research approach
Source: BMC Prim Care. 2026 Mar 30;27:184. doi: 10.1186/s12875-026-03299-1 (PMC13154494; doi:10.1186/s12875-026-03299-1)
Supplement: Supplementary file 2 — Supplementary Material 2. [file 12875_2026_3299_MOESM2_ESM.docx]

**Semi-structured Interview Guide (Rehabilitation Education)**

Date: ______ / ______ / ______  Study No.: __________________
Interviewer: _____________________

Years since graduation: ___________

Interview Questions

1. What does your typical work schedule look like?
2. What types of patients do you usually care for in outpatient clinics and home care settings?
    → *If home care is not mentioned:* “How about in home care?”
3. When interacting with patients and their families, do you consider any aspects beyond the medical perspective?
    → *If yes:* “Could you give some specific examples?”
4. In home medical care, have you ever been able to contribute from a rehabilitation perspective?
    → *If yes:* “What specifically did you do?”
    → “Was there anything you were unable to do?”
5. Have you experienced any hesitation or dilemmas when trying to incorporate a rehabilitation perspective as a physician?
    → *If yes:* “Could you describe the situation in more detail?”
6. Was there any content from the rehabilitation lectures that you were able to apply in your clinical practice?
    → *If yes:* “What specifically was helpful?”
7. Is there anything in the rehabilitation lectures that you would like to see improved or added?
8. Do you have any additional comments or questions?

*Thank the participant again and conclude the interview.*
